# Stem cell therapies in the clinic

**DOI:** 10.1002/btm2.70000

**Published:** 2025-02-04

**Authors:** Shrinivas Acharya, Suyog Shaha, Michael Griffith Bibbey, Malini Mukherji, Zongmin Zhao, Samir Mitragotri

**Affiliations:** ^1^ John A. Paulson School of Engineering and Applied Sciences Harvard University Massachusetts USA; ^2^ Wyss Institute for Biologically Inspired Engineering Harvard University Boston Massachusetts USA; ^3^ Department of Pharmaceutical Sciences, College of Pharmacy University of Illinois Chicago Chicago Illinois USA

**Keywords:** cell, cell therapy, clinical translation, clinical trials, HSC, iPSC, MSC, stem cell

## Abstract

Stem cell therapies have emerged as a transformative approach in modern medicine, with the potential to address and possibly cure a broad spectrum of diseases. These therapies utilize living stem cells that can perform complex biological functions not replicable by traditional drugs. Stem cell therapies have an expanding therapeutic landscape, with several products already approved and numerous clinical trials underway. Among the various stem cell types, hematopoietic stem cells (HSCs) and mesenchymal stem cells (MSCs) are most widely studied. In this review, we provide a detailed analysis of the current clinical landscape of stem cell therapies. We review 27 stem cell products that have received regulatory approvals and discuss 800 ongoing clinical trials, with a focus on HSCs and MSCs. We also discuss the critical challenges to be addressed to facilitate the clinical translation of stem cell therapies.

**Translational Impact Statement**

Over the past six decades, stem cell therapies have undergone significant advancements, with the field expanding through the development of diverse stem cell types, the incorporation of genetic engineering technologies, and the integration of biomaterial scaffolds. These innovations have significantly expanded the therapeutic potential of stem cells, paving the way for more precise and personalized treatments. This article presents a comprehensive review of the history and evolution of stem cell therapies, tracing clinical approval milestones, and highlighting ongoing clinical trials that are actively shaping the future of the field.

## 1 INTRODUCTION

Cell therapy refers to the injection, implantation or grafting of living cellular material inside the body to bring about a therapeutic effect.^1^ Unlike conventional therapeutic modalities such as small molecules, antibodies, and nucleic acids, cells can dynamically respond to biological cues, to offer multimodal actions and effectively penetrate biological barriers.^2^ In several instances, merely a single dose of therapeutic cells has been found to elicit long term curative responses.^3^ The practice of cell therapy dates back to the 19th century and continues to expand today, with a vast number of ongoing investigations.^1^ Cell therapies are currently a promising frontier of medicine.

The clinical landscape for cell therapies is primarily dominated by T cells and stem cells.^2^ T cells, which constitute the adaptive cellular immunity, were first utilized in adoptive transfer in 1986 to mount an antigen specific therapeutic response against cancer. Within three decades, T cell therapies have revolutionized cancer immunotherapy with multiple U.S. Food & Drug Administration (FDA)‐approved products for hematologic tumors and a recent approval for solid tumors in advanced melanoma patients.^4^ On the other hand, the use of stem cells as a cell therapy has a longer history. The remarkable ability of stem cells to regenerate themselves and differentiate into many different cell types render them capable of treating a diverse range of diseases. The first recorded marrow grafting in leukemia patients, aimed at preventing death from marrow failure post‐radiation, was published in 1959.^5^ Since then, significant developments in hematopoietic stem cell transplantation (HSCT) have been made. The isolation of another class of multipotent cell from bone marrow—mesenchymal stem cells (MSCs) in 1976 further opened up a new avenue for stem cell therapies.^6^ Since these seminal events, decades of continuous efforts in the field have led to the approval of stem cell products in various countries. Regulatory agencies in the United States, Europe, and Japan have also crafted their regulatory guidelines for validation of the safety and efficacy of stem cell products. Despite such promising advances, the vast majority of stem cell therapies are still in the early testing stage, with a rapid increase in clinical trials over recent years. These trials aim to either extend stem cell therapies to various untested diseases or address a particular challenge for manufacturing, safety or efficacy. In this review, we discuss the history and rapidly evolving clinical landscape of stem cell therapies.

**FIGURE 1:**
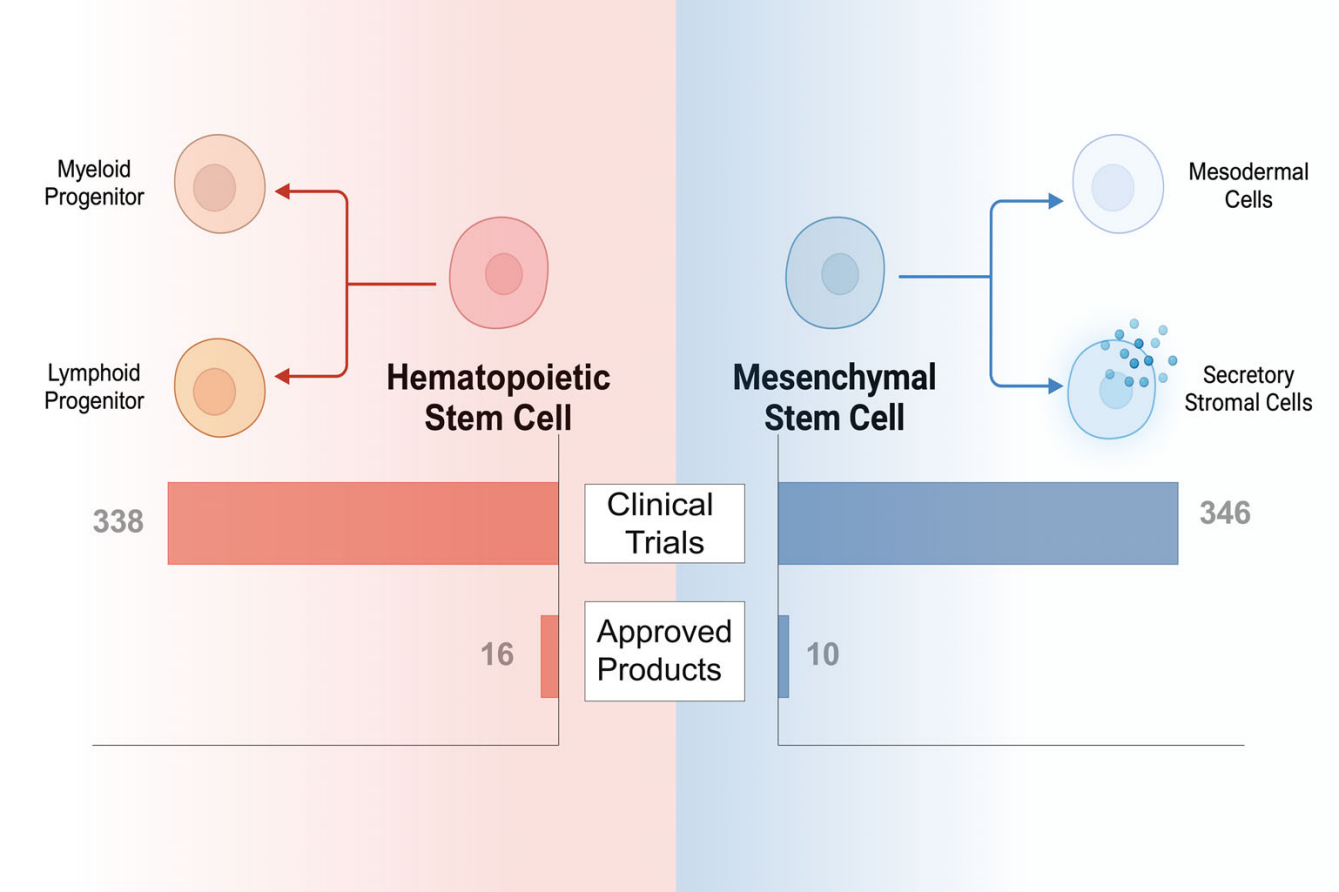
Common types of stem cells used in approved or clinically investigated stem cell therapies. HSCs and MSCs dominate the clinical landscape of stem cell therapies.

We first examined the historical efforts over the decades that have guided the development of stem cell therapies. Next, we identified 27 stem cell therapy products approved for clinical use in various countries and 800 active clinical trials utilizing stem cells as therapeutic modalities. Our analysis is limited to therapeutic interventions where stem cells are the primary component of the treatment, excluding trials and products that focus primarily on modalities other than stem cells. In our analysis, we found that two distinct types of stem cells—hematopoietic stem cell (HSC) and mesenchymal stem cell (MSC)—account for the majority of approved stem cell therapy products and clinical trials (Figure 1). Several specialized stem cells and non‐purified stem cell concentrates make up only a minority of therapies. Thus, we structure our discussion based on stem cell types. We provide a brief outline of historical developments, a comprehensive survey of approved products, a critical analysis of active clinical trials, and a detailed examination of the current challenges associated with stem cell therapies. This review is an update to our past analysis of cell therapies,^2^ with a sole focus on stem cell therapies. Additionally, we include stem cells administered as depot/scaffold systems, which were excluded from our past analysis,^2^ thus expanding our scope to cover tissue engineering applications.

## 2 BACKGROUND—BIOLOGY AND HISTORICAL DEVELOPMENT

Stem cells, present in both embryonic and adult tissues, possess remarkable abilities for self‐renewal and differentiation into various functional cell types.^7^ These cells are categorized based on their developmental potency, which indicates the diversity of cell types they can produce. Totipotent cells, such as the zygote, have the highest differentiation potential and can form all cell types of an organism, including extraembryonic tissues. Pluripotent cells, such as embryonic stem cells, can self‐renew and generate all cell types of the body but cannot form extraembryonic tissues.^7^ Multipotent cells, including hematopoietic and mesenchymal stem cells, can differentiate into cells within a specific lineage.^7^ Oligopotent cells, such as myeloid stem cells, have a more limited differentiation potential. Unipotent cells, such as spermatogonial stem cells, can differentiate into only one cell type.^7^ The use of zygote‐derived totipotent cells and embryonic stem cells is strictly restricted due to ethical issues, rendering them impractical for therapeutic use. There is extensive ongoing research focused on harvesting terminally differentiated cells from patients, converting them into induced pluripotent stem cells (iPSCs), and differentiating them into specialized cells that can be administered into the patients as therapies. In 2019, the first clinical trial of this kind using iPSC‐derived natural killer cells were tested in patients with cancer.^8^ Despite their potential, iPSC clinical studies are at its infancy and currently account for only a small number of clinical trials. In contrast, multipotent cells, such as HSCs and MSCs, make up the majority of current clinical trials. These will be the focus of this review, although other less common stem cell types will also be discussed.

### 2.1 Hematopoietic stem cells

HSCs are multipotent cells capable of differentiating into blood cells. Thus, HSC transplantation is well‐established for managing and potentially curing numerous hematologic diseases by reconstituting blood cells as a regenerative therapy or reprogramming the immune system as immunotherapy.^9^ Current HSCT procedures involve collecting HSCs from donors, purifying them, and transferring them to patients following preparative immunosuppressive conditioning. In certain cases, the purified HSCs are modified and expanded ex vivo before transplantation to enhance their therapeutic potential.^10^ Since the initial demonstrations of HSCT's feasibility and clinical utility, the protocol has been significantly refined to improve therapeutic outcomes and reduce transplant‐related complications.

The progress in HSCT began in the 1950s. Edward Thomas first introduced the procedure of allogeneic HSCT, transferring bone marrow cells from normal healthy donors to six patients in 1957.^10^ All patients in this trial died within 100 days post‐transplantation due to human leukocyte antigen (HLA) mismatching,^11^ which was discovered in 1969 as a critical factor for the safety of allogeneic HSCT.^12^ In the 1970s, the shortage of HLA‐matched donors remained a major bottleneck for HSCT, with only around 25% of patients in need having HLA‐matched stem cell donors.^13^ This challenge of HSCT was addressed with the development of stem cell isolation and cryopreservation protocols for autologous peripheral blood and allogenic umbilical cord blood. The first autologous HSCT was performed in humans in 1976, and the first umbilical cord blood allogeneic HSCT was conducted a decade later in 1988.^14^ Over time, these developments have enhanced HLA‐matching flexibility, improved HSC availability, and thus broadened the applicability of HSCT.

The emergence of HSCT in the 1980s enabled the treatment of cancer patients with higher doses of chemotherapy and radiation than previously established limits, significantly improving response rates, and in some cases, reducing mortality risk.^15^ However, many challenges remained regarding the purity of HSC product. The risk of contamination of harvested HSCs with malignant cells or pathogens increased the likelihood of disease relapse and post‐transplantation infections. Additionally, the presence of T cells or B cells in donor HSCs or the recipient's body elevated the risk of mixed chimerism and severe acute graft versus host disease (GvHD).^9^ To mitigate these risks, chemotherapy and total‐body radiation were developed as preparative and post‐transplant conditioning regimens. By controlling the underlying disease and eliminating the pre‐existing hematopoietic system, these regimens reduce graft rejection rates, immunodeficiency periods, and GvHD complications. The intensity of these conditioning regimens is tailored to patient's disease severity, age group, and existing comorbidities. These regimens are categorized into two types based on the intensity: Myeloablative conditioning (MAC) and reduced‐intensity conditioning (RIC). MAC eradicates the patient's immune cells and HSCs in the bone marrow to facilitate rapid donor HSC engraftment but has severe morbidities and organ toxicities. Conversely, RIC is used for older patients or those with comorbidities to minimize toxicities while providing sufficient immunosuppression to prevent donor HSC rejection. Currently, clinical investigators are personalizing regimens to strike the right balance between toxicity and immune ablation for successful HSC engraftment and effective disease control for each patient.^10^ The continuous development of conditioning regimens has widened the pool of potential HSC donors to include HSC‐matched unrelated donors or HLA‐haplotype matched donors, thereby increasing the chance of finding suitable matches. Additionally, advanced purification techniques for removing residual malignant cells and methods for depleting specific immune cells for isolated HSC have further reduced disease relapse and graft failures. The ongoing progress of antibiotics, antivirals and antifungals continues to improve infection control during HSCT.^16^

All these clinical advancements, including diverse stem cell sourcing options, HLA‐matching versatility from autologous to haploidentical donors, mild immunosuppressive conditioning regimens, highly purified CD34+ HSC formulations, and improved patient management, have significantly expanded the applicability of HSCT.^9^ Currently, HSCTs represent the highest number of approved cell therapy products for clinical use. The clinically recommended minimum dose for HSCT is 200–300 million cells per kg for optimal clinical outcome.^17^ The regenerative approach primarily utilizes autologous HSCT to minimize GvHD complications, while allogeneic HSCT is preferred for immunotherapeutic purposes, leveraging GvHD effects to maximize underlying disease control.^2^ The Stem Cell Therapeutic and Research Act of 2005 mandates all allogeneic HSCTs performed in the United States to be reported to the Stem Cell Therapeutic Outcomes Database (SCTOD). The Center for International Blood and Marrow Transplant Research (CIBMTR) manages this data and also collects voluntarily reported data for over 85% of autologous HSCTs performed in the United States.^18^ The analysis and ongoing trends based on this data are released annually by CIBMTR, providing a comprehensive understanding of real‐world clinical use of approved HSCTs.^19^ The accumulated clinical experience and knowledge over the past 75 years serve as a significant benchmark for further developing the HSCT approach.

### 2.2 Mesenchymal stem cells

MSCs are a distinct class of multipotent cells known for their ability to differentiate into various mesodermal cell types and their capacity to modulate the immune system. Consequently, MSCs have been extensively studied for both regenerative and immunomodulatory applications in clinical settings. Compared to HSCs, MSCs have a relatively shorter history in clinical research, spanning about four decades. The first infusion of MSCs in a patient was reported in 1995, marking the beginning of a broad exploration of their clinical potential.^20^

MSCs can be isolated from multiple sources, such as bone marrow^21^ and adipose tissue.^22^ They can also be obtained from traditionally discarded sources like umbilical cord tissue^23^ and placenta,^24^ which is advantageous for clinical translation. A typical clinical dose of MSCs, ranging from 100 to 150 million cells, can be isolated from just 25 mL of bone marrow aspirate.^25^ These cells can be readily grown and expanded in culture and have demonstrated the potential to differentiate into various mesenchymal tissue lineages including bone, cartilage, fat, and tendon under specific in vitro conditions.^26^

Despite the promising in vitro differentiation capabilities of MSCs, their in vivo applications have often encountered challenges such as suboptimal regenerative performance and low engraftment rates. However, therapeutic benefits have still been observed, primarily attributed to their stromal or tissue supportive properties.^27^ MSCs secrete a multitude of bioactive molecules that facilitate tissue regeneration.^28^, ^29^ Thus, even if they do not differentiate into the relevant cell type, they promote regeneration through anti‐apoptotic and trophic mechanisms.

In addition to their regenerative potential, another key mechanism for MSC‐based therapies is their ability to modulate immune responses. When primed with inflammatory factors like IFN‐γ, TNF‐α, IL‐1α, and IL‐1β, MSCs secrete numerous anti‐inflammatory factors. This activation process has paved the way for their use in conditions characterized by immune overactivation such as autoimmune diseases.^30^ However, it is important to note that the immunomodulatory activity of MSCs is not inherently active but is induced through a licensing process, necessitating specific pre‐conditioning protocols in therapeutic applications.^30^

Furthermore, the role of mechanotransduction in MSC differentiation and modulation has seen significant progress over the last 20 years. One of the seminal studies in this area was conducted by Engler et al. showing that the lineage differentiation of MSCs can be controlled by the elasticity of the substrate to which they are adhered.^31^ Further research by Chaudhuri et al. demonstrated that the viscoelastic properties of cellular microenvironment, such as the stress relaxation behavior of hydrogels, play a critical role in regulating MSC fate.^32^ These findings underscore the importance of the physical environment in optimizing MSC‐based therapies.

All these developments have fueled the continued use of MSCs in clinical investigations. Although MSCs have shown mixed efficacy in clinical settings, they have demonstrated an excellent safety profile. Their ability to differentiate and modulate immune responses, coupled with their safety profile, positions them as a versatile tool for treating a wide range of diseases.^25^

## 3 APPROVED PRODUCTS

**FIGURE 2:**
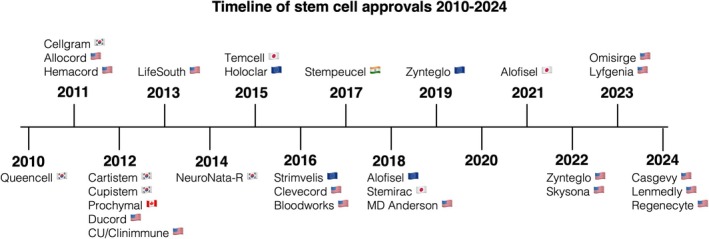
Timeline of regulatory approvals of stem cell products.

While stem cells have been utilized in the clinic for over 50 years, well‐defined, agency‐approved cellular products first became available in the 2010s. The first agency‐approved stem cell therapy was Queencell® (Anterogen), an MSC product for subcutaneous tissue defects, approved in 2010 by the South Korea Ministry of Food and Drug Safety (SK MFDS).^33^ In the following decade, the clinical adoption of stem cell therapy has expanded significantly, leading to many approvals from regulatory agencies worldwide (Figure 2). While this review focuses on the regulated use of stem cells in the clinic, it should be noted that there is widespread unregulated use of stem cells in both medical and cosmetic clinics globally. The FDA has issued consumer alerts regarding unregulated stem cell procedures.^34^

**FIGURE 3:**
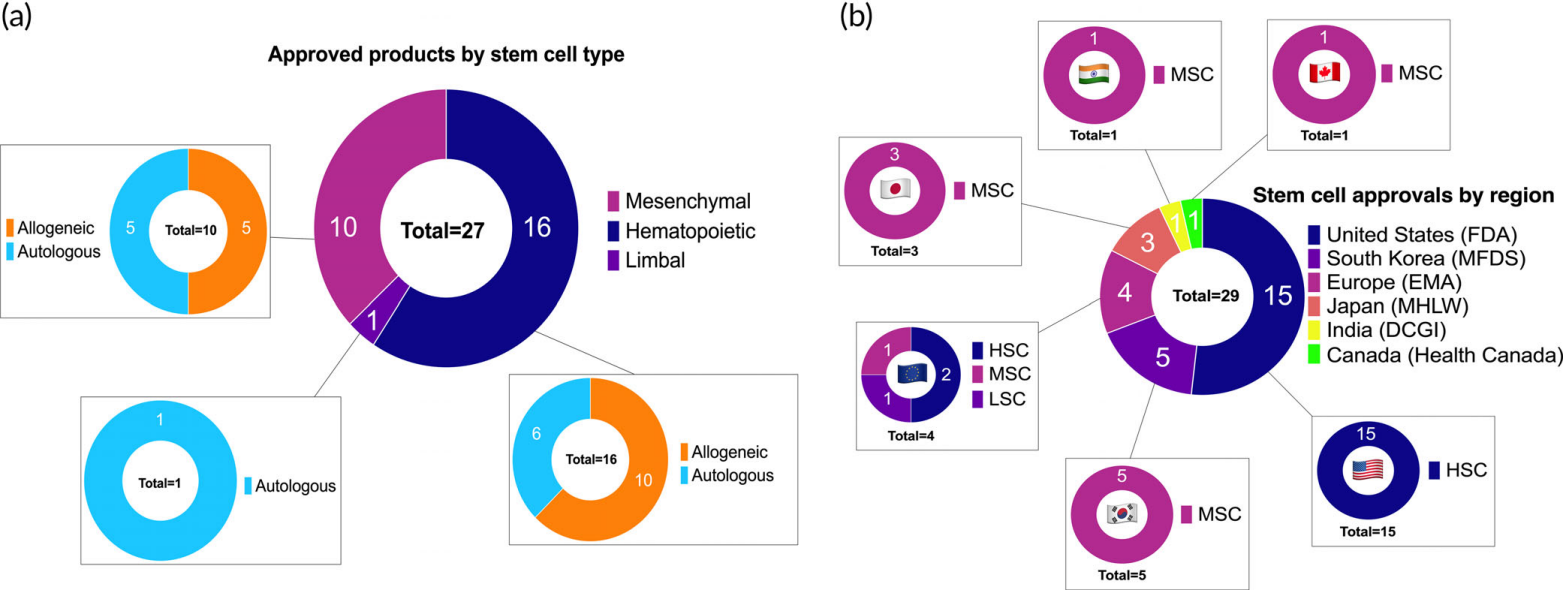
Analysis of approved stem cell therapy products. (a) Categorization of approved products by stem cell type. (b) Categorization of stem cell approvals by region.

According to our searches of literature and government databases, there have been 29 approvals for 27 stem cell products worldwide—comprising 16 HSC products, 10 MSC products, and 1 limbal stem cell (LSC) product (Table 1; Figure 3a). The variation in stem cell type, source, and approved indications seems to correlate with regional trends (Figure 3b).

**TABLE 1:** Clinically approved stem cell therapies, grouped by cell type.

Name; manufacturer	Isolation source	Cell source	Indications	Approval year (approval agency)	Genetically modified (yes/no)	Administration route	Dose (million cells/kg body weight)	Information source
HSC								
[Allocord] (HPC, Cord Blood); SSM Cardinal Glennon Children's Medical Center	Cord blood	Allogeneic	Hematopoietic system disorders	2011 (US FDA)	No	Intravenous	25	138
[Hemacord™] (HPC, Cord Blood); New York Blood Center	Cord blood	Allogeneic	Hematopoietic system disorders	2011 (US FDA)	No	Intravenous	25	139
[Ducord™] (HPC, Cord Blood); Duke University School of Medicine	Cord blood	Allogeneic	Hematopoietic system disorders	2012 (US FDA)	No	Intravenous	25	140
[N/A] (HPC, Cord Blood); Clinimmune Labs	Cord blood	Allogeneic	Hematopoietic system disorders	2012 (US FDA)	No	Intravenous	25	141
[N/A] (HPC, Cord Blood); LifeSouth Community Blood Centers	Cord blood	Allogeneic	Hematopoietic system disorders	2013 (US FDA)	No	Intravenous	25	142
[Clevecord™] (HPC, Cord Blood); Cleveland Cord Blood Center	Cord blood	Allogeneic	Hematopoietic system disorders	2016 (US FDA)	No	Intravenous	25	143
[N/A] (HPC, Cord Blood); Bloodworks	Cord blood	Allogeneic	Hematopoietic system disorders	2016 (US FDA)	No	Intravenous	25	144
[Strimvelis®]; Fondazione Telethon	Bone marrow	Autologous	ADA‐SCID	2016 (EMA)	Yes	Intravenous	2–20	145
[N/A] (HPC, Cord Blood); MD Anderson Cord Blood Bank	Cord blood	Allogeneic	Hematopoietic system disorders	2018 (US FDA)	No	Intravenous	25	146
[Zynteglo™] (betibeglogene autotemcel); bluebird bio	Peripheral blood	Autologous	TDT	2019 (EMA), 2022 (US FDA)	Yes	Intravenous	5	147
[Skysona®] (elivaldogene autotemcel); bluebird bio	Peripheral blood	Autologous	Slow down CALD progression	2022 (US FDA)	Yes	Intravenous	5	148
[Omisirge™] (omidubicel‐onlv); Gamida Cell	Cord blood	Allogeneic	Neutrophil recovery for hematological malignancies	2023 (US FDA)	No	Intravenous	92	149
[Lyfgenia] (lovotibeglogene autotemcel (lovo‐cel)); bluebird bio	Peripheral blood	Autologous	Sickle cell disease	2023 (US FDA)	Yes	Intravenous	3	150
[Casgevy] (exagamglogene autotemcel (exa‐cel)); Vertex Pharmaceuticals	Peripheral blood	Autologous	Sickle cell disease, recurrent VOCs, TDT	2024 (US FDA)	Yes	Intravenous	3	151
[Lenmedly] (atidarsagene autotemcel); Orchard Therapeutics	Peripheral blood	Autologous	Juvenile MLD	2024 (US FDA)	Yes	Intravenous	2–11.8	152
[REGENECYTE] (HPC, Cord Blood); StemCyte, Inc.	Cord blood	Allogeneic	Hematopoietic system disorders	2024 (US FDA)	No	Intravenous	25	48
MSC								
[Queencell®]; Anterogen	Adipose tissue	Autologous	Subcutaneous tissue defects	2010 (SK MFDS)	No	–	–	153
[Cellgram®]; Pharmicell	Bone marrow	Autologous	Acute myocardial infarction	2011 (SK MFDS)	No	Intravenous	Under 60 kg: 50 million total	154
							61–80 kg: 70 million total	
							81+ kg: 90 million total	
[Cartistem®]; Medipost	Cord blood	Allogeneic	Cartilage degeneration	2012 (SK MFDS)	No	Surgical or arthroscope	2.5 million/cm^2^ defect area	155
[Cupistem®]; Anterogen	Adipose tissue	Autologous	Crohn's fistula	2012 (SK MFDS)	No	Local intrafistular injection	30–60 million/cm fistula length	156
[Prochymal®] (remestemcel‐L); Osiris Therapeutics/Mesoblast Limited	Bone marrow	Allogeneic	Steroid‐refractory acute GvHD (pediatric)	2012 (Health Canada)	No	Intravenous	2	157
[NeuroNata‐R®] (lenzumestrocel); Corestem	Bone marrow	Autologous	ALS	2014 (SK MFDS)	No	Intrathecal	1	158
[TEMCELL® HS Inj.]; JCR Pharmaceutics	Bone marrow	Allogeneic	Acute GvHD following HSCT	2015 (JP MHLW)	No	Intravenous	2	159
[Stempeucel®]; Stempeutics	Bone marrow	Allogeneic	Critical limb ischemia due to Buerger's disease, knee osteoarthritis	2017 (DCGI)	No	Intralesional/Intramuscular	2	160
[Alofisel®] (darvadstrocel); TiGenix NV/Takeda	Adipose tissue	Allogeneic	Complex perianal fistulas in Crohn's disease	2018 (EMA), 2021 (MHLW)	No	Local intrafistular injection	120 million total	161
[Stemirac]; Nipro	Bone marrow	Autologous	Spinal cord injury	2018 (JP MHLW, conditional)	No	Intravenous	3.34	162
Other stem cells								
[Holoclar®]; Chiesi Farmaceutici/Holostem	Limbus (corneal edge)	Autologous	Limbal stem cell deficiency	2015 (EMA)	No	Surgical implantation	0.079–0.316 million/cm^2^	163

Abbreviations: ADA‐SCID, adenosine deaminase‐severe combined immunodeficiency; ALS, amyotrophic lateral sclerosis; CALD, cerebral adrenoleukodystrophy; DCGI, Drug Controller General of India; EMA, European Medicines Agency; GvHD, graft versus host disease; HPC, hematopoietic progenitor cell; HSCT, hematopoietic stem cell transplant; IR FDA, Iran Food & Drug Administration; JP MHLW, Ministry of Health, Labour and Welfare of Japan; MLD, metachromatic leukodystrophy; SK MFDS, Ministry of Food and Drug Safety of the Republic of Korea; TDT, transfusion‐dependent thalassemia; US FDA, U.S. Food & Drug Administration; VOCs, recurrent vaso‐occlusive crises.

### 3.1 Approved HSC products

Notably, 16 HSC‐based cell therapy products have been approved in the United States (Food and Drug Administration, FDA) and Europe (European Medicines Agency, EMA)—1 product by both agencies, 14 by only the FDA, and 1 by only the EMA. The FDA and EMA are the only agencies to formally approve HSC products, despite the widespread clinical practice of HSCTs worldwide. Data suggest that 1.5 million HSCT procedures have been performed globally between 1957 and 2019, with an increasing trend toward the use of allogeneic sources.^35^ The clinical prominence of HSCT continues to rise, as recent annual reports show more than 20,000 procedures in the United States and nearly 50,000 across Europe each year.^36^, ^37^ Importantly, transplant rates outside the United States and Europe are on the rise as well.^38^ For example, an analysis of HSCTs for acute myeloid leukemia shows that while high‐resource regions may have the greatest overall number of procedures, the sharpest increases are occurring in more resource‐limited regions, particularly in Africa and the Eastern Mediterranean.^39^ Given the global prominence of HSCT, it is important to note that not all approaches or practices utilize approved products or require agency approval. The FDA Regulatory Considerations for Human Cells, Tissues, and Cellular and Tissue‐Based Products outlines two primary criteria for regulation: minimal manipulation and homologous use.^40^ If donor tissue undergoes minimal manipulation (i.e., no novel methods or protocols involving cellular engineering, manufacturing, or other modification) and is transplanted to perform the same basic function (e.g., bone marrow tissue is used to replace, reconstitute, or supplement the hematopoietic system), the cells or tissue used do not require FDA approval.

However, recent advances in cellular engineering and manufacturing have led to a suite of HSC products that exceed minimal manipulation and require the quality control and safety standards of regulatory agencies. For example, Omisirge® (Gamida Cell) is a nicotinamide modified HSC product approved in 2023, where HSCs are isolated and expanded ex vivo with a proprietary nicotinamide treatment shown to enhance functionality.^41^, ^42^ Its noted improvements include reduced time to neutrophil recovery and a lower risk of infection.^41^, ^42^ Eight of the approved products are based on manufactured or preserved allogeneic cord blood with no modifications.^43^ In recent years, there have been several approvals for genetically modified HSCs. Zynteglo® and Lyfgenia® (Bluebird Bio) are treatments approved where autologous HSCs are isolated from patients and then transfected with lentiviral vectors to restore the beta‐globin gene.^44^, ^45^ These genetically modified HSCs are readministered intravenously to the patient to engraft to the bone marrow and produce functional red blood cells containing hemoglobin. Genetic engineering of HSCs has opened up innovative uses for HSC therapies beyond traditional HSCT (nonhomologous use). SKYSONA® (Bluebird Bio), another genetically modified HSC product, was approved in 2022 for treating cerebral adrenoleukodystrophy (CALD) in young boys.^46^ A similar lentivirus‐modified HSC treatment, Lenmedly™ (Orchard Therapeutics), was approved in 2024 for treating juvenile metachromatic leukodystrophy (MLD).^47^ REGENECYTE (StemCyte Inc.), a cord blood HSC therapy for hematopoietic and immunologic reconstitution, is the most recently approved HSC product, earning its approval in November 2024.^48^

### 3.2 Approved MSC products

No MSC products have been approved by the US FDA. However, outside the United States, MSCs are the most widely approved type of stem cell products, with 11 confirmed approvals for 10 different products. The products have been approved across South Korea (5), Japan (3), Europe (1), India (1), and Canada (1). A comprehensive summary of these products can be found in our previously published review.^2^ Asian countries represent 75% of all approved products. FDA approvals have been elusive largely due to inconclusive results in clinical trials. While stringent regulatory requirements related to MSC manufacturing, heterogeneity, safety, and dosing have posed significant challenges, the primary bottleneck limiting their approval in the US market is the poor efficacy observed in clinical trials.^49^ The disparity between the success of MSC therapies in preclinical animal models and their underperformance in human patients has been striking. Several potential pitfalls have been identified, including inadequate patient stratification, low MSC viability or fitness, and issues such as MSC trapping and clearance.^50^, ^51^ These challenges will be further discussed in a later section.

However, outside of the United States, MSC approvals have been granted for a variety of indications, highlighting the diverse potential of MSCs in cell therapy. We have identified four general categories where MSC products have garnered approval: soft tissue regeneration, osteoarthritis, central nervous system disorders, and GvHD. Products such as Queencell® (Anterogen), Cellgram® (Pharmicell), Cupistem® (Anterogen), Stempeucel® (Stempeutics), and Alofisel® (TiGenix NV/Takeda) are all approved for soft tissue indications. Two products are approved for osteoarthritis indications: Cartistem® (Medipost) and Stempeucel® (Stempeutics). Stempeucel® has dual marketing approvals in India for the treatment of critical limb ischemia and osteoarthritis of the knee. MSC products for central nervous system disorders encompass both neurodegenerative disease and spinal cord injuries. NeuroNata‐R® (Corestem) is approved for amyotrophic lateral sclerosis (ALS), and Stemirac (Nipro) for spinal cord injury. Finally, Prochymal® (Osiris Therapeutics/Mesoblast Limited) and TEMCELL® (JCR Pharmaceutics) have both been approved for acute GvHD.

There does not appear to be a definitive correlation between the source of approved MSC products, their therapeutic indications, or the regulatory agency responsible for their approval. Due to the limited number of approved products, it is difficult to assess whether cell source plays a key role in MSC approval. Six out of the ten approved MSC products are bone marrow‐derived, split evenly between autologous (Cellgram®, Stemirac, and NeuroNata‐R®) and allogeneic (Prochymal®, TEMCELL®, and Stempeucel®) sources. The approved bone marrow‐derived MSCs span all four therapeutic indication categories we identified. Adipose‐derived MSC products, including Queencell® (autologous), Cupistem® (autologous), and Alofisel® (allogeneic), are exclusively approved for soft tissue regeneration. However, bone marrow‐derived MSCs such as Stempeucel® (allogeneic) and Cellgram® (autologous), have also received approval for soft tissue indications. The only MSC product derived from allogeneic cord blood (Cartistem®) is approved for cartilage regeneration in osteoarthritis. Given the current landscape of approved products, assessing the role of tissue or donor source in the approval process remains limited and speculative. Cell source details are summarized in Table 1 and Figure 3.

### 3.3 Approved LSC products

Stem cells have been investigated in the clinic for use in ocular regeneration and ophthalmology since 1989, with significant progress made in the last 20 years.^52^, ^53^ The 2015 EMA approval of Holoclar (Chiesi Farmaceutici/Holostem) marked the first approval of a stem cell product in Europe.^54^ Holoclar is an autologous tissue engineered graft of limbal stem cells for use in limbal stem cell deficiency and corneal surface regeneration, particularly in cases of blindness caused by burning.^55^, ^56^ A tiny 1–2 square millimeter biopsy of undamaged limbal stem cells is cultured on human fibrin to produce a graft which can be transplanted into the damaged eye to regenerate a transparent, avascular cornea.^55^, ^56^

## 4 ACTIVE CLINICAL TRIALS

**FIGURE 4:**
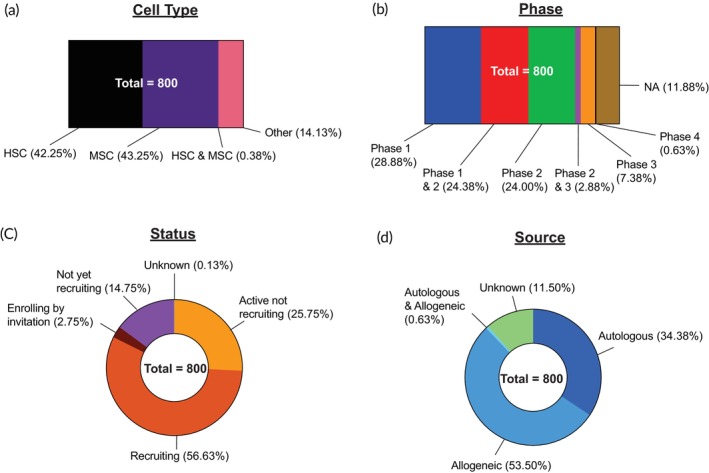
Overall landscape of stem cell clinical trials. Eight hundred stem cell clinical trials were analyzed and classified based on: (a) Type of cell; (b) Phase of trial; (c) Status of trial; and (d) Source of cell. Not Applicable (NA).

We collected active clinical trials of stem cell therapies through a systematic search of the “ClinicalTrials.gov” database. We used “stem cell,” “HSC,” or “MSC” as keywords. We selected interventional studies with the status “Not yet recruiting,” “Recruiting,” “Active, not recruiting,” “Enrolling by invitation” to identify active ongoing clinical trials. After manual screening, we identified 800 clinical trials in which stem cells were employed as the primary component of treatments. These 800 clinical trials were then analyzed in detail according to stem cell type (Figure 4a), trial phase (Figure 4b), trial status (Figure 4c), cell source (Figure 4d), administration method, and indications. It should be noted that for any disease indications that fall under more than one disease category, we only included them in one single category to avoid overcounting. For example, glioblastoma could be included in both the “Oncology” and “Neurology” categories. However, we have categorized it only under “Oncology.” We acknowledge the inconsistencies in such categorization during data analysis.

Clinical trials are conducted in different phases with each having a difference purpose: Phase 1—to investigate the safety and side effects of the treatment in a small group of people (20–80); Phase 2—to determine the effectiveness of the treatment in a larger group of people (100–300); Phase 3—to confirm the effectiveness, monitor side effects, and compare the treatment with an established standard; Phase 4—to track the safety and seek more information about the drug after its approval.^57^

Of the 800 active trials analyzed, there is an almost equal number of trials for HSCs (*n* = 338, 42.25%) and MSCs (*n* = 346, 43.25%). In terms of cell source, 53.50% are allogeneic, while 34.38% are autologous. A detailed analysis based on cell type is presented below. Representative examples of trials are shown in Table 2.

**TABLE 2:** Representative examples of current clinical trials of stem cell therapies, grouped by cell type.

Trial number	Cell source	Genetic modification	Indication	Administration route	Phase
HSC (*n* = 338)					
NCT03981549	Autologous	_	Central retinal vein occlusion	Intravitreal	1, 2
NCT03866109	Autologous	Lentiviral vector driving IFN‐α2 expression	Glioblastoma multiforme	Intravenous	1, 2
NCT00977691	Allogeneic	_	Sickle cell anemia	Intravenous	1, 2
NCT04390971	Autologous	BCL119 gene modified by CRISPR‐Cas9	Transfusion dependent beta‐thalassaemia	Intravenous	NA
NCT05863845	Autologous	_	Diffuse large B cell lymphoma	Intravenous	NA
NCT01560182	Autologous	ARSA cDNA introduced by means of 3rd gen VSV‐G pseudotype lentiviral vectors	Lysosomal storage disease	Intravenous	1, 2
NCT03615105	Allogeneic	_	Leukemia	Intravenous	2
NCT05662904	Unknown	CRISPR/Cas‐9 mediated CD33 deletion	Acute myeloid leukemia (AML)	Intravenous	1
NCT05815004	Autologous	_	Gaucher disease	Intravenous	2, 3
NCT03367546	Allogeneic	_	Inherited metabolic disorders	Intravenous	2
NCT05401162	Autologous	_	Ovarian cancer	Intravenous	NA
NCT01515462	Autologous	Lentiviral vector encoding Wiskott‐Aldrich syndrome (WAS) protein	Wiskott–Aldrich syndrome (WAS)	Intravenous	1, 2
NCT04482413	Autologous	_	Alzheimer disease	Intravenous	2
NCT04849065	Autologous	_	Amyotrophic lateral sclerosis (ALS)	Intramuscular	2
NCT05595681	Allogeneic	_	Diabetic foot ulcer	Subcutaneous dermoepidermal junction injection	1
NCT05797272	Autologous	_	Hemoglobin Bart's hydrops	In utero transplantation	NA
NCT03707262	Allogeneic	_	Renal transplant rejection	Intravenous	1, 2
HSC and MSC (*n* = 3)					
NCT03724136	Autologous	_	Brain diseases	Intravenous and intranasal	NA
NCT02582775	Allogeneic	_	Epidermolysis bullosa	Intravenous	2
MSC (*n* = 346)					
NCT04074408	Unknown	_	Basal ganglia hematoma	Intracavitary injection	2
NCT05165628	Allogeneic	_	Diabetic foot ulcer	Dressing	1
NCT02808208	Autologous	_	End stage renal disease	Topical application	1, 2
NCT05705024	Allogeneic	_	Corneal ulcer	Subconjunctival injection	2
NCT05909488	Allogeneic	_	Retinitis pigmentosa	Peribulbar injection	2, 3
NCT01652209	Unknown	_	Acute myocardial infarction	Intracoronary injection	3
NCT04925024	Allogeneic	_	Hypoplastic left heart syndrome	Intramyocardial injection	2
NCT05456243	Allogeneic	_	Kidney transplant	Intra‐arterial	1
NCT05003908	Allogeneic	_	Diabetes	Intravenous	1
NCT04888949	Unknown	_	SARS‐CoV‐2 (COVID‐19)	Intravenous	2
NCT04097652	Allogeneic	_	Acute ischemic stroke	Intravenous	1
NCT03570450	Allogeneic	_	Stroke	Intravenous	1
NCT03550183	Unknown	_	Parkinson's disease	Intravenous	1
NCT05018767	Allogeneic	_	Frailty	Intravenous	1
NCT04314661	Allogeneic	_	Osteoarthritis	Intra‐articular injection	1, 2
NCT03467919	Autologous	_	Osteoarthritis	Intra‐articular	3
NCT04118088	Allogeneic	_	Crohn's disease	Injection into fistula	4
NCT04018729	Unknown	_	Chronic obstructive pulmonary disease severe	Endoscopic administration	2, 3
NCT05854641	Allogeneic	_	Critical limb ischemia	Intramuscular	4
NCT04088058	Autologous	_	Liver cirrhosis	Intrahepatically	2
NCT05167721	Autologous	_	Multiple system atrophy	Intrathecal	2
NCT03876197	Autologous	_	Xerostomia	Intraglandular transplantation	1, 2
NCT03298763	Unknown	Genetically modified to express TRAIL (TNF‐related apoptosis inducing ligand)	Adenocarcinoma of lung	Unknown	1, 2
Other stem cells (*n* = 113)					
NCT05139056	Unknown	_	Recurrent glioblastoma	Intracerebral injection	1
NCT02592330	Autologous	_	Limbal stem cell deficiency	Transplanted into cornea	1, 2
NCT03763136	Allogeneic	_	Heart failure	Intramyocardial injections	1, 2
NCT05063721	Autologous	_	Mitochondrial myopathies	Intra‐arterial	1
NCT03879876	Allogeneic	_	Severe combined immunodeficiency (SCID)	Intravenous	1, 2
NCT03952273	Unknown	_	Coronary artery disease	Local implant	NA
NCT04728906	Autologous	_	Myocardial infarction	Heart patch	NA

### 4.1 HSC clinical trials

#### 4.1.1 Emerging trends in active clinical trials

**FIGURE 5:**
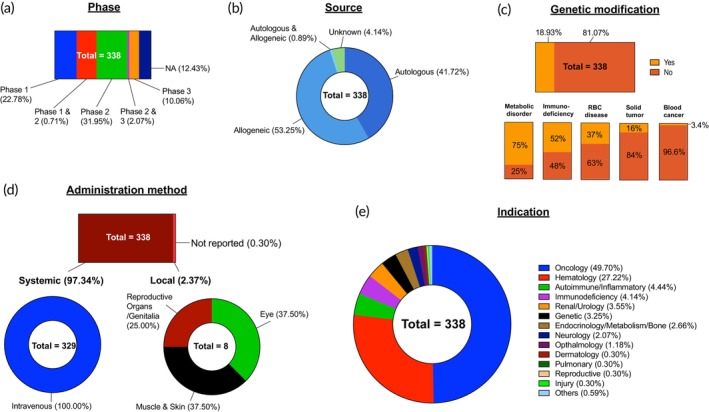
Landscape of HSC active clinical trials. Three hundred and thirty‐eight HSC clinical trials were analyzed and classified based on: (a) Phase of trial; (b) Source of cell; (c) Genetic modification; (d) Administration method, which was further classified into systemic and local routes; and (e) Indication type. Not applicable (NA).

HSCs are utilized in 42.3% (*n* = 338) of the clinical trials analyzed. Among these trials, 44% are in Phase 1, 34% in Phase 2, and only 10% in Phase 3 (Figure 5a). Despite the well‐established use of HSCs in treating a variety of diseases, the predominance of early‐phase trials indicates significant interest in advancing HSCT into next‐generation therapies. These early trials include investigations into the synergy of new agents with the HSCT procedure. Pre‐transplant combinations are exploring optimal agents for depleting specific immune cells such as T cells and B cells, while post‐transplant combinations aim to leverage clinically available immunomodulatory agents to control underlying diseases and support engraftment during the acute post‐transplant phase. These agents include preparative immune‐depleting antibodies like aCD3 (e.g., NCT00319657) and aCD45 (e.g., NCT03670966), ex vivo T‐cell/B‐cell depleting devices (e.g., NCT03653338, NCT02061800), and post‐transplant immunomodulators like PD1 inhibitor (e.g., NCT05137886), aCD38 (e.g., NCT04268498, NCT04965155), aCD33‐drug conjugate (e.g., NCT02221310, NCT02117297), aCD19 (e.g., NCT03333187, NCT02790515), aCD319 (e.g., NCT02420860, NCT03030261), T cells (e.g., NCT05239676, NCT05632380), NK cells (e.g., NCT05654038, NCT05250362), and dendritic cell vaccines (e.g., NCT03334305, NCT03396575). This extensive interest in new combinations appears to be driven by recent findings that selective depletion of adaptive immune cells such as B and T cells from donor HSC products and recipient patients during the preparative stage can prevent GvHD complications in both acute and chronic stages. This approach reduces the need for post‐transplant immunosuppressive treatments and allows for the use of mild or even non‐toxic conditioning regimens in HSCT, thereby significantly improving patients' quality of life.^58^

Conversely, some early‐stage trials are exploring innovative applications of HSCT, such as addressing depression in psychiatric disorders stemming from alcohol addiction (e.g., NCT03265808), inducing donor organ tolerance in lung (e.g., NCT03500731) and kidney transplants (e.g., NCT00319657, NCT00619528), replacing the immune system with genetically modified HSCT capable of targeting specific markers in solid tumors (NY‐ESO‐1; e.g., NCT03240861, NCT03691376), evading human immunodeficiency virus (HIV) infections (anti‐HIV genes; e.g., NCT02797470, NCT01961063), imparting resistance to chemotherapy (P140K MGMT; e.g., NCT05052957), delivering cytokines in a tumor‐specific manner (e.g., NCT03866109), and repairing damaged retinal tissue in ischemic or degenerative conditions (e.g., NCT03981549, NCT01736059, NCT04925687). These trials aim to expand HSCT's applicability to new indications, reflecting the field's enthusiasm for advancing beyond traditional uses. Among the HSCT trials, 10% are in Phase 3. Of these, 82% focus on improving HSCT procedures for conditions such as blood cancers, metabolic disorders, and genetic blood disorders, where some HSCT products are already clinically approved. Notably, the remaining 18% of Phase 3 trials investigate HSCT for broader applications, including treatment of solid tumors such as eye cancer (NCT00554788), brain cancer (NCT00085202, NCT00336024, NCT00653068), and breast cancer (NCT01646034), as well as reducing the risk of organ rejection in kidney transplantation (NCT03363945). These trials evaluating HSCT typically focus on primary outcomes related to toxicity, efficacy, or both. Toxicity outcomes include measures such as the severity grades of graft‐versus‐host disease, incidence of dose‐limiting toxicities, and treatment discontinuation rates. Efficacy outcomes typically assess metrics such as graft rejection rates, engraftment rates, minimal residual disease negativity, relapse rates, progression‐free survival, overall response rates, and discontinuation due to the need for subsequent secondary treatments. The primary outcomes investigated in HSCT trials vary significantly depending on the trial phase, target disease, and scope of the investigation, making it difficult to map and analyze trends systematically.

#### 4.1.2 Source of HSC therapies

In our analysis, we found that trials involving allogeneic HSCT (53.3%) slightly outnumber those involving autologous HSCT (41.7%) (Figure 5b). This is in stark contrast to current clinical practice, where autologous HSCTs are still performed more frequently than allogeneic HSCTs.^19^ However, with the growing number of unrelated donors registering for HSC donation, allogeneic HSCTs may soon surpass autologous HSCTs in clinical settings.^59^ Unlike most other cell therapies, the choice of donor type in HSCT not only has logistical implications but also significantly impacts therapeutic outcomes. Allogeneic HSCs, once transplanted, can elicit a therapeutic response by targeting malignant cells in the recipient's body through the alloimmune effector mechanisms, such as the graft‐versus‐host reaction.^60^ This reaction can produce pro‐immunogenic effects in cancer patients or anti‐immunogenic effects in autoimmune diseases, making allogeneic HSCT potentially more beneficial for these conditions.^61^, ^62^ In blood cancer treatment, where the primary goal is to eradicate cancerous immune cells and prevent relapse, 63.3% of HSCT trials utilize allogeneic donors. Conversely, for applications aimed at rescuing patients from the toxic effects of high‐dose chemotherapy or radiation therapy or rectifying a patient's blood system affected by acquired or congenital disorders, autologous HSCT, which is well‐tolerated, holds greater promise. In these cases, the graft‐versus‐host reaction has minimal therapeutic significance. Notably, 90.6% of HSCT trials that involve ex vivo genetic engineering use autologous cells, as this approach is often employed to correct genetic defects in a patient's own HSCs. Interestingly, 87.5% of HSCTs used for treating solid tumors also utilize autologous cells. This is likely due to the modest benefits observed with the graft‐versus‐tumor effect of allogeneic HSCs in most solid tumors during the early 2000s, which led to a decline in their use.^63^ Currently, the primary goal of HSCT in solid tumors is to mitigate the toxic effects of cancer treatments.

In addition to donor type, the tissue source of HSCs is also crucial in HSCT. Clinically, the three main sources are bone marrow stem cells, harvested mainly through multiple punctures and aspirations of the iliac crest; mobilized stem cells, collected via leukapheresis of peripheral blood; and cord blood stem cells, obtained from the umbilical cord at the time of childbirth. However, many trials do not specify the source tissue, making it challenging to assess its impact. Based on available clinical data, peripheral blood is generally preferred for adult recipients due to its avoidance of painful bone marrow collection and the faster engraftment post‐transplantation. This preference is expected to grow as issues related to GvHD, stemming from the higher presence of adaptive immune cells in peripheral blood HSCs compared to bone marrow, are increasingly mitigated. Although bone marrow collection, which was more commonly used in earlier times, now accounts for a minority of transplants, it remains predominantly used for pediatric recipients in both allogeneic and autologous HSCT. This is likely due to the challenges of administering granulocyte‐colony stimulating factor (G‐CSF) and performing leukapheresis in children, as well as the lower risk of chronic GvHD and non‐relapse mortality associated with bone marrow compared to peripheral blood HSCs.^64^ Cord blood is currently the least commonly used HSC source, mainly because of its low HSC yield and the slower hematologic and immune reconstitution it provides, often necessitating the use of multiple umbilical cord units for a single recipient.^62^

#### 4.1.3 Disease indications

The specifics of HSCT procedure are based on the disease of interest. For example, 19% of active HSCT trials use genetically engineered HSCs, however, this number differs significantly based on the disease type (Figure 5c). We discuss these aspects in detail.

Around 82.5% of HSCT trials focus on blood‐related disorders, including blood cancers, autoimmune and immunodeficiency diseases, and genetic blood abnormalities (Figure 5e). This focus is also reflected in the approved HSC products, primarily due to HSCs' ability to rectify and reset the hematopoietic system. All these HSCT trials exclusively use intravenous administration of HSCs (Figure 5d). This method is favored because HSCs, regardless of the donor type or source tissue, naturally home to bone marrow niches once introduced into the bloodstream. Notably, only about 10% of infused HSCs successfully engraft within the marrow microenvironment during a typical HSCT,^65^ and increasing this engraftment rate could enhance the hematopoietic system reconstitution.^66^

Blood cancer accounts for 53% of HSCT trials targeting blood‐related disorders. Since HSCT is a well‐established treatment for blood cancers, most of these trials explore variations in the pre‐ or post‐transplant conditioning regimens. Notably, 3.4% of the HSCT trials for blood cancer involve genetically engineered HSCTs (Figure 5c). These include trials investigating the incorporation of anti‐HIV genes into donor HSCs using lentiviral vectors to make transplanted cells resistant to HIV infection in HIV‐related lymphoma (e.g., NCT01961063, NCT02337985, NCT02797470) and trials using the CRISPR‐Cas9 technology to knockout the CD33 gene in HSCs to make them resistant to anti‐CD33 drug treatment in acute myeloid leukemia (e.g., NCT05662904, NCT04849910).

The next major focus of HSC trials for blood‐related disorders is non‐malignant diseases associated with red blood cells (RBCs), accounting for 33% of trials. These primarily include genetic abnormalities in RBCs, such as sickle cell disease, anemia, and thalassemia. HSCT represents a promising treatment option for these disorders, providing the potential for lifelong benefits with a single intervention. In these trials, 37% involve genetically engineered HSCs (Figure 5c), all of which are autologous. In contrast, the remaining trials for these diseases that do not involve genetic modifications predominantly use allogeneic HSCs. Autologous HSCT with gene‐modified cells avoids major immunological complications associated with allogeneic HSCT, thereby increasing the chances of curing patients with genetic RBC disorders.^67^ However, the complications and technical challenges of achieving therapeutically sufficient genetic engineering of autologous HSCs sustain interest in developing non‐genetic allogeneic HSCTs.^67^ A few allogeneic HSCT trials aim to avoid immune mismatch‐related toxicities and elicit curative outcomes by performing in‐utero HSCT for treating congenital hematologic disorders diagnosed early in pregnancy (e.g., NCT02986698, NCT05797272). These approaches leverage the normal development of the fetal immune system during ontogeny to facilitate successful allogeneic HSC engraftment without serious mismatch‐related toxicity.^68^ Except for these two trials, all other HSCT trials for RBC disorders use intravenous administration.

Another major focus of HSCT trials related to blood‐related diseases involve conditions associated with the overactivation or underdevelopment of the immune system, accounting for 14% of the remaining trials. These include autoimmune diseases such as multiple sclerosis, Crohn's disease, and systemic lupus, where the goal is to re‐establish immuno‐tolerance, and various genetic immunodeficiency diseases, where the aim is to correct genetic abnormalities and reset the immune system to a healthy state. None of the HSCT trials for autoimmune diseases involve genetic modification of HSCs, and they exclusively use intravenous infusion of HSCs. Non‐genetic approaches primarily rely on high dose lymphoablative conditioning to achieve curative benefits for autoimmune diseases. Lymphoablation effectively deletes the immune memory by clearing autoreactive B and T cells from the body, which are then restored by non‐genetic HSCT. This explains why 80% of these trials use autologous HSCs, as they can provide better immune restoration compared to allogeneic HSCs. The remaining trials using allogeneic HSCs aim to employ mild‐conditioning regimens that preserve the patient's immune system while leveraging allogeneic HSCT enriched with tolerance‐promoting cells (NCT00497952, NCT02674217). This approach seeks to establish a state of mixed chimerism, significantly reducing toxicities associated with conditioning and transplantation, and decreasing the frequency of infections by maintaining the patient's immune system throughout the HSCT period. The absence of genetic engineering in HSC trials for autoimmune diseases is due to conflicting results in preclinical studies. These studies found that HSCs modified with xenogenes to induce specific tolerance against self‐antigens responsible for autoimmunity led to significant inflammation, priming the immune system against the transplanted cells and failing to induce sufficient tolerance.^69^, ^70^ Preclinical exploration of immunosuppression strategies is ongoing to reduce inflammation and aid in the induction of better tolerance with HSCs modified with xenogenes.^71^ For immunodeficiency disorders, which are predominantly genetic, 52% of HSCT trials involve genetic engineering to modify HSCs (Figure 5c). Similar to HSCT trials for genetic disorders of RBCs, all these trials involving genetically modified HSCs to address defects in white blood cells (WBCs) exclusively use autologous HSCs. In these trials, the modified cells are infused intravenously. Conversely, HSCT trials that do not involve genetic engineering of HSCs exclusively use allogeneic sources, similar to the approach used for genetic disorders related to RBCs. This similarity indicates that the approach to correcting defective genes with HSCs is independent of whether the genetic defect affects RBCs or WBCs. Both sets of genetic problems in vastly different components of the hematopoietic system can be addressed by HSCT using a similar therapeutic approach. This versatility makes HSCT a suitable treatment for a diverse range of blood‐related disorders, as is actively studied in clinical trials.

The remaining 17.5% of HSCT trials cover a broad range of indications, including solid tumors, organ transplantation, metabolic disorders, dermatological diseases, infections and psychiatric disorders (Figure 5e). Solid tumor indications account for the majority of these HSCT trials (37.5%). All these trials involve intravenous injection of HSCs, with 87.5% using autologous cells. This preference is because the primary goal of HSCT in solid tumors is to restore the patient's hematopoietic and immune systems, which are often damaged by the high‐dose radiotherapy or chemotherapy used to eradicate solid tumor cells. Notably, 21% of these HSCT trials are in Phase 3, all aiming to achieve this restoration with HSCT.

Brain cancer represents a major focus of HSCT trials for solid tumors, accounting for 40% of these trials, with 30% in Phase 3. In addition to providing regenerative benefits by engraftment in the bone marrow, HSCT has been shown to home to brain tumors post‐intravenous administration in preclinical studies.^72^, ^73^ This potential is driving the interest in using HSCT for brain tumor treatment, although the exact homing mechanism remains unknown. Only 16% of trials for solid tumors involve genetic modification of HSCs (Figure 5c). For example, one trial involves overexpressing a mutant methylguanine methyltransferase (MGMT) promoter with a chemoresistance gene to provide chemoprotection during high‐dose MGMT inhibitor chemotherapy (NCT03240861). Another trial aims to limit IFN‐α2 gene expression in post‐transplant HSC‐derived myeloid cells to brain tumors, reducing non‐tumor secretion of IFN‐α2 (NCT03866109). Other trials involve genetically modifying HSCs to express NY‐ESO‐1 T cell receptor (TCR) for treating ESO‐1+ solid tumors (NCT03691376, NCT03240861), aiming to restore the immune system with cells that can specifically attack and kill ESO‐1+ cancer cells, thereby halting disease progression or relapse. All these clinical trials using genetic modification for HSCT in solid tumor treatment are in early phases and exclusively use autologous HSCs.

The second major focus of HSCT trials for non‐blood related disorders is solid organ transplantation, accounting for 22% of trials. The primary goal in these applications is to induce tolerance to transplanted organs by injecting HSCs from the same donor at the time of organ transplantation. Preclinical studies have shown that this approach significantly improves long‐term outcomes by reducing chronic alloreactivity to the transplanted organ and minimizing the non‐specific side effects of lifelong immunosuppressive drug use.^74^ By replacing the recipient's immune system with the donor's, this method allows for the acceptance of the transplanted organ as their own. Of these trials, 92% focus on kidney transplantation, while 8% involve lung transplantation. Since replacing blood cells is the main goal, all trials use intravenous infusion of HSCs, with almost all deriving HSCs from organ transplantation donors, making them allogeneic for the HSC recipient. Furthermore, 92% of these trials are in early phases. As the understanding of the mechanisms to induce tolerance with HSCs grows, HSCT in combination with solid organ transplantation is likely to evolve, potentially broadening its applications and increasing organ availability for patients with end‐stage organ failure.

The next major focus of HSCT trials related to non‐blood related disorders is for treating metabolic disorders, accounting for 20.3% of trials. This group mainly includes lysosomal storage disorders related to lysosomal metabolism, Fabry disease related to fat metabolism, and Hurler's syndrome related to sugar metabolism. These metabolic disorders are primarily genetic. All these trials use intravenous infusion of HSCs, with 93% in early phases. Of these trials, 75% employ genetic modification of autologous HSCs (Figure 5c), while the remaining 25% use unmodified allogeneic HSCs, following a pattern similar to trials for blood‐related genetic disorders. Currently, all genetic modifications in these trials are performed with lentiviral vectors, though the CRISPR technology may soon emerge as the primary method of gene modification. In these ways, HSCs are being investigated for treating a diverse array of non‐blood disorders in various clinical trials, representing great promise for the upcoming years.

### 4.2 MSC clinical trials

**FIGURE 6:**
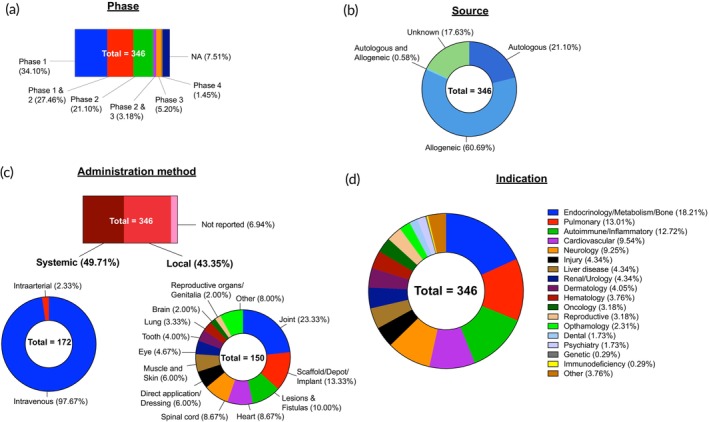
Landscape of MSC active clinical trials. Three hundred and forty‐six MSC clinical trials were analyzed and classified based on: (a) Phase of trial; (b) Source of cell; (c) Administration method, which was further classified into systemic and local routes; and (d) Indication type. Not applicable (NA).

A total of 346 trials utilize MSCs as a therapeutic entity. The majority of these trials are in the early phases, with 34.10% in Phase 1, 27.46% in Phase 1/2, and 21.10% in Phase 2. Only a small fraction of trials have progressed to Phase 3 (5.20%) (Figure 6a). The Phase 3 trials are investigating a variety of indications, including COVID‐19 (NCT05689008, NCT05682586), osteoarthritis (NCT05086939, NCT04427930, NCT03467919), and myocardial infarction (NCT01652209, NCT05043610, NCT05935423). Additionally, five MSC‐based products are undergoing Phase 4 trials (NCT05854615, NCT05854641, NCT05660824, NCT04675359, NCT04118088). Notable examples include Stempeucel® (intramuscular injection at a dose of 200 million cells) for treating critical limb ischemia and Darvadstrocel (direct injection into the fistula at a dose of 150 million cells) for treating complex perianal fistula.

#### 4.2.1 Source of MSCs

The majority (60.96%) of the MSC clinical trials involve allogeneic MSCs (Figure 6b). This preference may stem from two key reasons: (1) expanding and characterizing off‐the‐shelf allogeneic human MSCs is less restrictive compared to autologous cells; (2) although a debated topic, MSCs have long been considered hypoimmunogenic or “immune‐privileged,” leading clinicians to favor the logistically advantageous and readily available allogeneic sources. However, there have been studies describing immune rejection and the generation of antibodies against allogeneic donor MSCs, challenging the “immune‐privileged” notion.^75^ While it is challenging to definitely determine which source is superior, two trials (NCT05086939, NCT05789719) are comparing autologous versus allogeneic MSCs for treating knee osteoarthritis. Results from such trials may provide useful guidance on selecting suitable MSC sources for specific or a more general class of indications.

#### 4.2.2 Delivery methods

Unlike HSCs, there is no universal delivery route for MSCs across different diseases. The administration routes for MSCs are almost equally distributed between local delivery (43.35%) and systemic delivery (49.71%) (Figure 6c). For optimal efficacy, the delivery method depends on the specific indication and therapeutic mechanism.^76^ Systemic delivery, which includes intravenous and intra‐arterial administration, has been employed for various indications such as COVID‐19, autoimmune diseases, neurological diseases, and liver diseases. Intravenous delivery is the most popular systemic delivery method due to its ease of administration and excellent safety profile.^77^ However, the non‐specific distribution of cells throughout various organs and tissues following intravenous delivery may lead to off‐target effects.^78^

Among local delivery methods, intra‐articular administration (23.33%) is the most commonly used, particularly for osteoarthritis. Due to the very low vascularity of the articular cartilage, systemic delivery is ineffective.^79^ Other indications involving the brain and spinal cord, such as ALS, cerebral palsy, and spinal cord injuries, are being treated in clinics through intrathecal injection (8.67%) of MSCs. Additionally, direct delivery to the heart via intracoronary and intramyocardial injections (8.67%) has been reported in clinical studies for cardiovascular indications. Intracoronary injections are less traumatic than intramyocardial injections, but the use of specialized equipment during intracoronary injections, such as over‐the‐wire (OTW) angioplasty balloon carriers, can potentially lead to vascular injury.^80^ Another advantage of intramyocardial injections is their higher cell engraftment rate in the myocardium as compared to intracoronary injections.^81^ Intramuscular injection constitutes approximately 6% of trials, with preclinical mouse models demonstrating the additional benefit of increased dwell time.^82^ Moreover, the secretome of intramuscularly delivered MSCs has been shown to exhibit systemic effects by entering the circulation.^83^

Better understanding of cell‐material interaction, particularly for MSCs, has motivated the use of biomaterial‐based technologies in the clinic. Of the total trials involving local MSC delivery, 13.33% are using either a depot/implant or a scaffold‐based technology, while 6.00% are employing some form of dressing or topical application containing MSCs (Figure 6c). These biomaterial technologies are generally used for treating musculoskeletal abnormalities and ulcers. For example, the use of allogeneic umbilical cord‐derived MSCs in hyaluronate hydrogel (Cartistem) has shown promising efficacy in osteoarthritic patients for cartilage regeneration.^84^ In patients with complete spinal cord injury, supraspinal control of movements was regained following the implantation of NeuroRegen (Collagen) scaffolds with human umbilical cord MSCs.^85^ Tricalcium phosphate is a commonly used material in combination with MSCs for bone defect or spinal fusion applications.^86^, ^87^, ^88^ Human placenta‐derived MSCs cultured in electrospun gelatin nanofibrous scaffolds have been used to accelerate wound healing in patients with diabetic foot ulcers.^89^ The support of MSCs with amniotic membrane has also been explored in clinical trials for wound healing applications.^90^ Hydrogel sheets of MSCs have been in clinical trials for treatment of diabetic foot ulcers.^91^

#### 4.2.3 Disease indications

The immunomodulatory and regenerative properties of MSCs have significantly broadened their clinical utility (Figure 6d). The largest category of indications falls under the endocrinology/metabolism/bone bracket (18.21%), which primarily include diabetes‐related conditions and osteoarthritis (Figure 6d). Intravenous infusion of MSCs has been used to treat type 1 diabetes, shifting serum cytokine patterns from pro‐inflammatory to anti‐inflammatory and increasing the number of regulatory T cells in peripheral blood cell. This approach has been shown to reduce daily insulin requirements, lower hemoglobin A1c (HbA1c) levels, and improve C‐peptide levels.^92^ MSCs have also demonstrated therapeutic effect in clinical trials for type 2 diabetes, leading to reductions in insulin usage and glycated hemoglobin levels.^93^, ^94^, ^95^ Additionally, intra‐articular injection of MSCs to osteoarthritic patients has resulted in improved knee function, reduced inflammation, and regeneration of hyaline‐like articular cartilage.^96^, ^97^, ^98^

Autoimmune/inflammatory indications comprise 12.72% of the total MSC trials, primarily addressing Crohn's disease, sclerosis, COVID‐19 related acute respiratory distress syndrome (ARDS) and GvHD (Figure 6d). Upon intravenous injection, MSCs first home to the lungs and exert strong systemic and local immunomodulatory effects on the host immune system.^99^ There are 29 trials focused on COVID‐19‐related indications, leveraging the immunomodulatory property of MSCs to manage the overactivation of the immune system. Several meta‐analyses of clinical trials have shown that MSCs can lower the risk of mortality in COVID‐19 patients by reducing inflammation and improving pulmonary function.^100^, ^101^ Another eight trials are investigating the use of MSCs for GvHD, capitalizing on their immunomodulatory capabilities. MSCs have also demonstrated therapeutic clinical responses following HSCT or in cases of hematologic malignancy, particularly in acute GvHD.^102^

Cardiovascular indications comprise 9.54% of the total MSC trials. MSCs have shown to increase left ventricular ejection fraction in patients with heart failure.^103^ Pulmonary indications account for 13.01% of the total trials (Figure 6d). Additionally, there are 11 trials focused on indications related to sexual dysfunction or infertility. Other less common indications being explored in MSC trials include xerostomia (e.g., NCT03876197), dry eye syndromes (e.g., NCT03878628), Pitts Hopkins syndrome (e.g., NCT05165017), leukocyte adhesion defect (e.g., NCT05162326), allergic rhinitis (e.g., NCT05167552), septic shock (e.g., NCT04961658), AIDS (e.g., NCT05872659), and hepatitis B (e.g., NCT03826433).

Genetically engineered MSCs are rarely used in the identified MSC trials. In the entire pool of MSC trials, there is only one genetically modified product (NCT03298763), which is currently in the Phase 1/Phase 2 stage. In this trial, MSCs are genetically modified to express TNF‐related apoptosis‐inducing ligand (TRAIL) ligand for the treatment of non‐small cell lung cancer in combination with cisplatin/pemetrexed chemotherapy.

### 4.3 Other stem cell clinical trials

**FIGURE 7:**
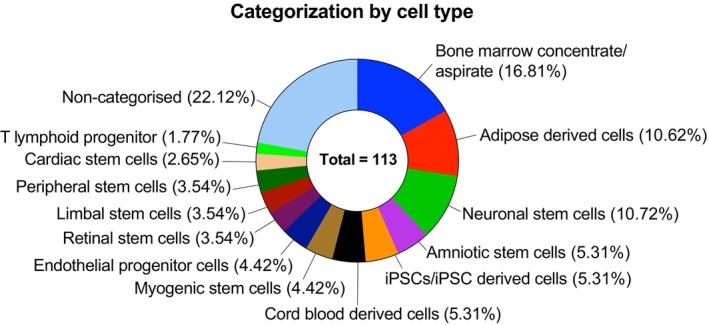
Current landscape of other stem cell clinical trials. One hundred and thirteen trials were analyzed and categorized based on the specificity of stem cell.

There are a total of 113 (14.13%) trials involving other stem cell types beyond purified HSCs or MSCs. These trials involve crude concentrates like bone marrow aspirate, adipose‐derived cells, and cord blood, which are obtained without isolation, as well as cells specific to one lineage like neuronal stem cells, retinal stem cells, limbal stem cells, cardiac stem cells, and induced pluripotent stem cells (iPSC) derived cells (Figure 7). There are only 47 trials with these specialized stem cells, which may be due to: (1) their limited differentiation potential and functional diversity as compared to HSCs and MSCs,^104^ (2) difficulty in sourcing and manufacturing these specific cell types at a therapeutic dose.^105^ The eye is a major target for stem cell therapy due to its easy accessibility, immune‐privileged environment, and the relatively small number of cells required for transplantation.^106^ For this reason, limbal stem cells (four trials) have been used to address limbal stem cell deficiency (e.g., NCT03957954), while retinal stem cells (four trials) have shown promise for the treatment of macular degeneration (e.g., NCT05187104). For applications for repairing damaged central nervous system (e.g., NCT03684122), neural stem cells (12 trials) have proved to be a promising option. Similarly, cardiac stem cells (3 trials) are being investigated for treatment of various heart conditions (e.g., NCT03406884), due to their therapeutic potential through paracrine and regenerative mechanisms.^107^ Endothelial progenitor cells (5 trials), known for their superior angiogenic and tissue repair capabilities, are being investigated for various applications, such as myocardial infarction (e.g., NCT00936819) and liver cirrhosis (e.g., NCT03109236).^108^ Most of these trials are in early phases, with a few having reached Phase 3. For example, a Phase 3 trial (NCT02781922) is investigating intracoronary delivery of cardiac stem cells for treating hypoplastic left heart syndrome.

Interestingly, in this category, local delivery (~65%) is the most commonly used delivery method, which differs from the trends seen in HSC and MSC clinical trials. Direct delivery to the heart through intramyocardial or intracoronary injections of cardiac stem cells or iPSC‐derived cardiomyocytes has been explored in more than 10 trials. Similarly, delivery to the brain through intracerebral or intracranial injections of neural stem cells has been investigated in clinical trials. Systemic delivery is generally used for the crude concentrates mentioned above.

## 5 MAIN CHALLENGES FOR CLINICAL TRANSLATION OF STEM CELL THERAPIES

**FIGURE 8:**
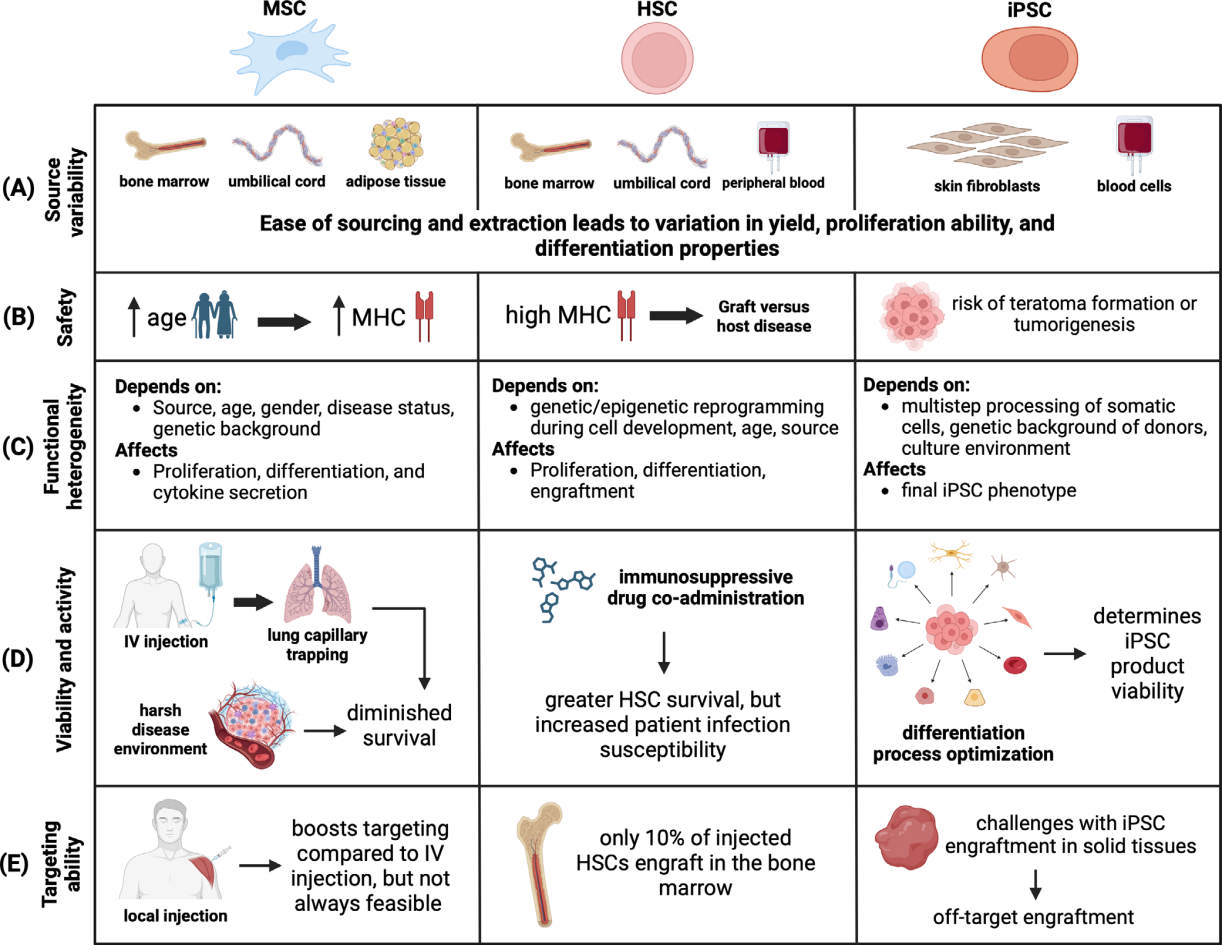
Challenges for clinical translation of stem cell therapies. For MSC, HSC, and iPSC cell types, various challenges were identified and categorized based on: (a) Source variability; (b) Safety; (c) Functional heterogeneity; (d) Viability and activity; and (e) Targeting ability.

Despite significant advances in both preclinical and clinical settings, several major challenges should be addressed before stem cell therapies can be fully implemented in the clinic. Some of the key considerations include: (i) identifying viable stem cell sources, (ii) addressing safety and immunogenicity concerns, (iii) managing functional heterogeneity, (iv) ensuring biological activity and viability post‐transplantation, and (v) optimizing targeted delivery and migratory capacity (Figure 8). Our discussion in this section primarily focuses on these challenges as they relate to widely used stem cells, such as MSCs and HSCs, while also examining specific issues associated with iPSCs, which are emerging as a promising new type of stem cell‐based therapies.

### 5.1 Viable stem cell sources

To understand the challenges of stem cell transplantation in vivo, it is crucial to consider how sourcing varies between cell types and how it affects therapeutic efficacy. The primary sources of MSCs in the clinic are bone marrow, adipose tissue, and umbilical cord (Figure 8a). The source plays a key role in determining the number of MSCs that can be harvested, their proliferation ability, and their differentiation potential. Historically, bone marrow was the major source of MSCs, but harvesting them requires highly invasive procedures, which carry risks of infection and pain. Additionally, only around 0.001%–0.01% of the isolated mononuclear cells from bone marrow are MSCs, and these cells have limited proliferation ability.^109^ Adipose tissue, on the other hand, has become a more advantageous source for MSCs, as they can be isolated with much less invasive procedures and yield 500 times more MSC precursors than an equivalent amount of bone marrow.^27^ Despite this advantage, adipose tissue‐derived MSCs tend to differentiate primarily into adipose tissue, limiting their use in generating other desired tissues. Umbilical cord blood‐derived MSCs make up only 0.2%–1.8% of the isolated mononuclear cells.^109^ MSCs derived from umbilical cord blood exhibit better proliferation ability than bone marrow‐derived MSCs and can differentiate into bone, cartilage, and adipose tissue. Age is also a major consideration when isolating autologous MSCs, as older age is associated with senescence and reduced proliferation and differentiation capabilities of MSCs. Variations in donor characteristics such as gender, tissue source, and disease status can also affect MSC quality and therapeutic efficacy. Allogeneic MSCs can help address some of these challenges, but the same donor characteristics will influence their effectiveness. Additionally, variability in MSC isolation methods from clinic to clinic make it difficult to standardize procedures across the board. To address the inconsistencies arising from the use of MSCs from different sources and isolation procedures, the International Society for Cell & Gene Therapy (ISCT) set a minimum standard for defining human MSCs in 2006.^110^

HSCs can be isolated from umbilical cord blood, bone marrow, and peripheral blood (Figure 8a). It has been reported that at least 2 × 10^8^ bone marrow cells per kg of body weight are needed to achieve efficient transplantation and reconstitution.^111^ The ex vivo expansion of isolated HSCs is essential for generating a sufficient number of cells for transplantation. However, HSCs face challenges such as limited growth potential, tendency to differentiate when cultured, and loss of stemness over time. To address these limitations, protocols are being developed to utilize self‐renewal regulators like Homeobox protein‐B4 (HOXB4), angiopoietin‐like proteins (ANGPTL), and Follistatin‐like 1 (FSTL1), as well as various cytokines and growth factors like IL‐6, fms like tyrosine kinase 3 (Flt3), IL‐3, and thyroid peroxidase (TPO) to control HSC self‐renewal, enhance ex vivo expansion, maintain multipotency, increase progenitor survival, and boost proliferation.^111^, ^112^

iPSCs can be sourced from a variety of tissues, but they are primarily derived from skin fibroblasts and blood cells (Figure 8a).^113^ Despite the reduced immune response and personalized nature of autologous iPSC sources, allogeneic sources are currently preferred due to time and financial considerations. Like other autologous stem cell therapies, iPSCs face similar time‐consuming challenges, as the process of producing autologous cell products is lengthy, making them impractical for treating certain acute indications such as heart failure.^114^ Several methods to mitigate the risk of immune rejection following allogeneic cell transplantation are being developed, such as the development of HLA haplotype banks and “HLA cloaking”—a process that involves inactivating or suppressing MHC expression in iPSCs.^114^, ^115^ The use of iPSCs raises unique ethical concerns related to their production. Although iPSCs address ethical issues associated with human embryonic stem cells by enabling the reprogramming of adult somatic cells into any desired cell type, their infinite differentiation potential poses additional ethical dilemmas. These include concerns about human reproductive cloning, genetic modification of embryos, the production of human gametes, and the creation of interspecies chimeric animals.^116^, ^117^ Although human cloning is prohibited worldwide, recent advancements such as the cloning of macaque monkeys via somatic cell nuclear transfer,^118^ highlight the potential for human cloning, emphasizing the need for stringent regulations on iPSC use. Genetic modification brings along concerns regarding the safety, precision, and reproducibility of the modified cells, particularly for their implantation in humans, necessitating the need for strict adherence to GMP guidelines and quality control measures. Production of human gametes also raises ethical concerns, including the lack of consent from donors and the potential for unauthorized reproduction. Lastly, interspecies chimeric animal organ production and testing also bring up unique ethical questions.^117^

### 5.2 Safety and immunogenicity

Safety risks associated with the immunogenicity of many stem cell therapies pose significant challenges to treatment efficacy. MSCs are unique in that they exhibit low immunogenicity due to their low expression of MHC/HLA I and the lack of MHC/HLA II markers.^110^ However, allogeneic MSCs do not have complete immune privilege. MHC‐I mismatches between donor and recipient, especially with increasing donor age, can lead to increased immune rejection (Figure 8b). Autologous MSCs can avoid MHC mismatch, but they can carry genetic defects from the patients, which can impair their efficacy and therapeutic potential. Additionally, as MSCs are typically administered intravenously, there is a risk of an instant blood‐mediated inflammatory reaction (IBMIR), an innate immune attack that can negatively impact cell engraftment, viability, and overall therapeutic of MSCs.

HSCs are much more prone to immune rejection than MSCs due to their high expression of MHC (Figure 8b). This can result in GvHD, where either the recipient's immune system attacks the donor's cells or the donor's cells attack the recipient's organs. Typically, before administering HSCs, patients undergo total body irradiation or chemotherapy to lower the risk of GvHD and create space in the patient's bone marrow for donor HSC engraftment. However, less than 25% of patients can tolerate this pre‐treatment due to its intensity, which can sometimes lead to mortality.^119^ Additionally, these conditioning treatments are non‐specific, which can result in complications affecting various organ functions, the need for red blood cell and platelet transfusions, secondary malignancies, infertility, and prolonged immune suppression, leaving patients vulnerable to infections. Safer alternatives, such as antibody‐mediated treatment using a CD117 antibody‐drug conjugate linked to saporin, allow for targeted depletion of HSCs and offer a more controlled and less risky method of preparing the bone marrow niche.^119^ Gene therapy also presents a promising solution by enabling in situ editing of HSCs, avoiding the risks of conditioning treatments and allogeneic transplantation.^120^

The risk of teratoma formation is the most significant safety concern associated with iPSC therapy in the clinic (Figure 8b).^121^ While the unlimited proliferative capacity of iPSCs is advantageous for therapeutic applications by enabling the large‐scale production of cell products, it can also poses a risk due to the high tumorigenic potential from differentiation into off‐target cell type.^114^ To mitigate this risk, robust and highly efficient methods of directed differentiation of iPSCs into the target cell product must be established to minimize the presence of undifferentiated cells in the final product. Moreover, rigorous purification strategies can be employed to eliminate unwanted cells. These include the use of chemotherapeutic drugs capable of eliminating rapidly proliferating cells, antibodies selective for mature and undifferentiated cells, and karyotyping to identify genetic abnormalities. Advanced strategies such as incorporating suicide genes that trigger apoptosis in unwanted cell types can also help ensure the safety of the final iPSC‐derived product.^121^, ^122^, ^123^, ^124^

### 5.3 Functional heterogeneity

The functional heterogeneity of stem cells arises from a multitude of factors including tissue source, donor characteristics, and intercellular differences within tissues. MSCs' properties vary heavily depending on their source as well as the donor's age, gender, disease status, and genetic background (Figure 8c). These variations in tissue sources and donor profiles lead to differences in the proliferation and differentiation capabilities of MSCs, resulting in varying levels of therapeutic effectiveness between treatments. For example, umbilical cord blood derived MSCs have higher immunomodulatory potential, bone marrow MSCs are more effective at promoting regeneration, and adipose‐derived MSCs show greater adipogenic differentiation and produce more cell matrix components. Cytokine secretion is another important property of MSCs, as it helps regulate tissue repair and modulate the overall immune response, though this also varies depending on the tissue source and donor. For example, adult adipose‐derived MSCs show higher pro‐inflammatory cytokine secretion compared to younger adipose‐derived MSCs, which reduces their immunomodulatory capacity.^110^ Donor characteristics play a key role in the properties of MSCs. MSCs obtained from male donors exhibit higher metabolic activity and greater proliferation ability, whereas MSCs derived from female donors possess a stronger osteogenic response.^125^ Additionally, the health status of the donor impacts MSC functionality. For example, MSCs from patients with advanced osteoarthritis show reduced proliferative ability and decreased chondrogenic and adipogenic differentiation abilities compared to those from healthy donors. Age is another critical factor, as increasing donor age has been associated with declines in both proliferative and differentiation abilities of donor MSCs.^126^

The heterogeneity of HSC function can stem from various actors, including differences in genetic and epigenetic reprogramming during cell development and maturation, variable localization within bone marrow niches, and genetic changes driven by molecular and cellular stimuli (Figure 8c). Aging also plays a major role in the functionality of HSCs, with increased myelopoiesis and decreased lymphopoiesis as cells age. Additionally, the engraftment efficiency of HSCs in the bone marrow after transplantation varies depending on the donor's age and the stem cell source. HLA matching is one of the most important factors in evaluating donors for HSC transplantation, but matching gender and choosing a young donor age are equally important considerations. It has been shown that donors over the age of 40 are associated with a significantly higher risk of acute and chronic GvHD as well as increased transplant‐related mortality.^127^ Additionally, male recipients of HSCs from female donors may experience higher rates of non‐relapse mortality and GvHD.^128^

iPSCs tend to exhibit greater heterogeneity than other stem cells due to the multistep reprogramming process required to generate them from somatic cells, which can introduce experimental variations (Figure 8c). Additionally, genetic differences between individuals, along with alterations that occur during reprogramming, can lead to significant changes in iPSC phenotypes, such as differentiation capacity, morphology, and epigenetic profiles. For example, a genomic analysis led by the Human Induced Pluripotent Stem Cells Initiative of 711 iPSC lines from 301 healthy donors found that genetic differences among donors accounted for 5%–46% of the variability in iPSC phenotypes.^129^ Thus, genetic analysis is a crucial step to validate the homogeneity of iPSC‐derived products and to identify any genetic or experimentally‐induced heterogeneity.

### 5.4 Maintenance of biological activity and viability after transplantation

In any cell therapy, it is crucial that the transplanted cells maintain viability and retain their function. Unfortunately, stem cell therapies face many challenges in this regard.

The injection route for MSCs greatly affects their viability and ability to perform their desired functions in vivo (Figure 8d). Intravenous (IV) injection is the clinical standard, but many studies have shown that IV‐injected cells often become trapped in lung capillaries and do not survive more than a few days.^82^ Additionally, many MSCs die upon encountering the hostile environment of the disease site.^130^ The viability of MSCs post‐transplantation can also depend on the cryopreservation and thawing protocols used. While optimal protocols are still being developed, research shows that culturing cells overnight after thawing before injection significantly increase MSC survival. Recent studies indicate that intramuscular injection of MSCs can extend their survival to more than 5 months in situ, compared to only a few days with IV injection and 3–4 weeks with intraperitoneal or subcutaneous injections.^82^ Various solutions are being explored to improve MSC viability and function after transplantation, including priming MSCs with hypoxia, cytokines, or small molecules, as well as using hydrogel scaffolds, microgel‐mediated delivery, and surface modification of the cells.^131^

The successful transplantation of HSCs and their subsequent viability and activity depend on many factors such as the conditioning regimen, the source of the cells, age and disease states of the donor and recipient, and co‐administration of immunosuppressive drugs (Figure 8d). The strength of the conditioning regimen significantly impact the engraftment rate of HSCs in the bone marrow. Within the bone marrow niche, interactions between HSCs and their non‐stem cell neighbors are essential to maintaining the balance between quiescent and self‐renewal, ensuring the stability of the HSC pool.^132^

For iPSCs, optimizing their differentiation into the desired cell product is critical for therapeutic efficacy after delivery (Figure 8d). For example, preclinical studies on iPSC‐derived cardiomyocytes have shown that engraftment efficiency depends on the duration of the differentiation process, with a 20‐day period yielding the highest efficiency.^133^ Thus, the fabrication process for mature cell product should be optimized to minimize off‐target effects post‐delivery and maximize therapeutic potential.

### 5.5 Targeted delivery and migratory capacity

The ability of transplanted cells to home to the desired site in the body is critical for therapeutic efficacy. Therefore, it is important to understand where stem cells naturally traffic and how targeted delivery varies with different delivery methods.

In clinical trials, MSCs are locally delivered in 49% of cases, highlighting the preference for controlled delivery to the disease site when possible, as opposed to systemic administration (Figure 8e).^130^ As previously mentioned, systemic administration of MSCs through IV injection is hindered by the fact that many MSCs become trapped in lung capillaries, and IBMIR further limit their homing ability. The percentage of IV‐administered MSCs that successfully reach the target site is in the low single digits. As a result, most MSC therapies that are in late‐stage clinical trials rely on local administration methods such as intrathecal, endocardial, or intralesional injections. However, local administration is not always feasible, as many indications require more invasive delivery methods. To improve MSC homing, several approaches are being explored, including increasing the expression of CXCR4, a chemokine receptor that plays a key role in cell migration,^134^ as well as using magnetic guidance, cell surface engineering, ex vivo priming, among other methods.^135^

HSCs are primarily administered to home to the bone marrow and rebuild its microenvironment. However, only about 10% of administered HSCs successfully engraft in the bone marrow (Figure 8e).^65^ The source of HSCs also affects engraftment rates, with umbilical cord blood HSCs exhibiting greater delays in engraftment compared to those derived from bone marrow or peripheral blood. Intra‐bone administration is being explored as an alternative to the standard IV administration in order to deliver HSCs directly to the bone marrow and lower the risk of GvHD. However this approach can also damage the bone marrow microenvironment, limiting its effectiveness.^136^

iPSCs face significant delivery challenges in achieving tissue engraftment in solid tissues. Systemic infusion of iPSC‐based therapies is generally insufficient to establish grafts in target organs and can lead to off‐target engraftment (Figure 8e).^137^ To address these issues, bioengineered scaffolds are being developed to support iPSCs and allow for direct transplantation into the desired organ.^122^ However, these methods still require invasive procedures, which may limit their applicability.

## 6 CONCLUSION

Stem cells, with their inherent ability to function as living systems, offer therapeutic potential far beyond that of traditional treatments. Numerous stem cell therapies have gained approval from regulatory agencies globally, demonstrating remarkable success in treating a wide range of diseases. Efforts are ongoing to expand the use of these therapies into new therapeutic areas, reflecting the growing versatility of these treatments. At the same time, advancements in cellular biology are driving the development of novel therapies, broadening the scope of what stem cell therapies can address. However, despite these advances, the field still faces significant challenges, particularly in areas related to the biology, manufacturing, and regulation of these therapies, all of which need to be addressed to facilitate wider clinical adoption. Overcoming these obstacles will require a concerted effort involving further biological research, the establishment of standardized clinical protocols, and the development of innovative manufacturing processes. Despite these challenges, the continued success of stem cell therapies in clinical settings ensures that this area of research will remain a hub of innovation, with more transformative therapies likely to emerge in the near future.

## AUTHOR CONTRIBUTIONS

**Shrinivas Acharya:** Data curation; formal analysis; investigation; visualization; writing – original draft; writing – review and editing. **Suyog Shaha:** Data curation; formal analysis; investigation; visualization; writing – original draft; writing – review and editing. **Michael Griffith Bibbey:** Data curation; formal analysis; investigation; visualization; writing – original draft; writing – review and editing. **Malini Mukherji:** Data curation; formal analysis; investigation; visualization; writing – original draft; writing – review and editing. **Zongmin Zhao:** Conceptualization; funding acquisition; writing – review and editing. **Samir Mitragotri:** Conceptualization; writing – review and editing; funding acquisition.

## CONFLICT OF INTEREST STATEMENT

SM and ZZ are inventors on patent applications in the field of cell therapies (owned and managed by Harvard University).

## Data Availability

The data that support the findings of this study are available from the corresponding author upon reasonable request.
